# Supplementary material for: Cerebral Vasospasm as a Critical Yet Overlooked Complication Following Tumor Craniotomy: A Systematic Review of Case Reports and Case Series
Source: J Clin Med. 2025 Apr 1;14(7):2415. doi: 10.3390/jcm14072415 (PMC11989413; doi:10.3390/jcm14072415)
Supplement: Supplementary file 1 [file jcm-14-02415-s001.zip › jcm-3400254-supplementary.pdf]

Supplementary Table S1. Characteristics of studies included.

Author	Year	Age	Sex	Pathology	Location	Vascular Encasement	Surgery	Complication	
								Intraoperative	Post Operative
Afshari *et al*	2014	9	F	Hypoglossal Schwannoma	Skull base, Middle cranial fossa	ACA	TSS	ND	ND
Aoki *et al*	1995	51	F	Cavernous Malformation	Skull base, Middle cranial fossa	ND	Craniotomy	Bleeding	Hydrocephalus,
		46	M	Adenocarcinoma	Skull base, Middle cranial fossa	ND	Radical Craniofacial Resection	Bleeding	ND
		16	F	Craniopharyngioma	Skull base, Middle cranial fossa	ND	Partial Removal Of A Tumor Arising From The Hypothalamus;	ND	ND
		60	M	Meningioma	Skull base, Middle cranial fossa	ACA	Craniotomy	ND	ND
Bejjani *et al*	1999	48	F	Meningioma	Skull base, Middle cranial fossa	ICA	ND		ND
		65	F	Meningioma	Skull base, Posterior cranial fossa	VBA	Total Tumor Resection With Extreme Lateral Transcondylar Approach	ND	Epidural hematoma
		50	F	Meningioma	Skull base, Middle cranial fossa	ICA	ND	ND	ND
		37	M	Chordoma	Skull base, Posterior cranial fossa	VBA	ND	ND	ND
		33	M	Schwannoma	Skull base, Middle cranial fossa	ICA	ND	ND	ND
		57	M	Meningioma	Skull base, Anterior cranial fossa	BILAT ICA/VBA	ND	ND	ND
		59	F	Meningioma	Skull base, Middle cranial fossa	ICA	ND	ND	ND
		50	F	Meningioma	Skull base, Anterior cranial fossa	ND	ND	ND	ND

		38	F	Meningioma	Skull base, Middle cranial fossa	ICA	ND	ND	ND
Bougaci et al	2017	60	M	Pituitary Micro Adenoma	Skull base, Middle cranial fossa	ND	TSS	ND	Subarachnoid bleeding
Budnick et al	2020	36	F	Meningioma Suprasellar	Skull base, Middle cranial fossa	PCA Sx, B Supraclinoid ICA Dan A1	TSS	ND	Diabetes insipidus, Nasal CSF Leak
		55	M	Pituitary Adenoma	Skull base, Middle cranial fossa	ND	TSS	CSF Leak	Subarachnoid bleeding In Basal Cisterna Diabetes insipidus
Campt et al	1980	33	F	Sellar Adinoma	Skull base, Middle cranial fossa	ND	TSS Hypophysectomy	CSF Leak	ND
Cervoni et al	1996	51	M	Suprasellar Adenoma	Skull base, Middle cranial fossa	ND	TSS	Lacerated of Chiasmatic Cistern	A Collection Of Blood In The Chiasmatic And Interpedicular Cisterns
		48	F	Sellar-Episellar Adenoma	Skull base, Middle cranial fossa	ND	Craniotomy	ND	Collection Of Blood In The Chiasmatic Cistern
		38	M	Craniopharyngioma	Skull base, Middle cranial fossa	ND	Craniotomy	ND	A Collection Of Blood In The Chiasmatic Cistern,
		44	M	Adenocarcinoma	Skull base, Anterior cranial fossa	ND	Craniotomy	ND	A Collection Of Blood In The Sylvian Fissure
		51	M	Meningioma	Skull base, Posterior cranial fossa	ND	Craniotomy	Copious Bleeding	Collection Of Blood In The Supratentorial Cisterns,
Ecker et al	2003	23	F	Ruptured Dermoid Cyst	Skull base, Anterior cranial fossa	ND	Craniotomy	ND	ND
Esenou et al	2016	43	F	Pituitary Adenoma	Skull base, Middle cranial fossa	ND	TSS	No Complication	Base Tumor Bleeding
Hashikata-hirokoni et al	2023	70	M	Large Pituitary Adenoma	Skull base, Middle cranial fossa	ND	TSS	ND	Hidrocephal
Hyde-rowan et al	1983	30	F	Pituitary Macroadenoma	Skull base, Middle cranial fossa	ND	TSS	Intracavernous Site Bleeding	CSF Leak
Kasliwal et al	2008	34	F	Pituitary Macroadenoma	Skull base, Middle cranial fossa	ND	TSS	CSF Leak	Bleeding In Suprasellar Cistern

Leurox et al	1991	69	F	Neuroma Accoustic	Skull base, Middle cranial fossa	ND	Craniectomy	ND	Subdural Effusion Bilateral
		42	M	Meningioma	Skull base, Middle cranial fossa	L External Carotid Artery	Craniotomy	ND	Accumulation Of Blood In The Basal Cisterns
Mansouri et al	2012	29	F	Pleomorphic Atypical Aggressive Adenoma	Skull base, Middle Cranial Fossa, Anterior And Posterior Cranial Fossa	ND	TSS	ND	Subarachnoid bleeding
		43	F	Aggressive Adrenocorticotropic Hormone Cell Adenoma	Skull base, Middle cranial fossa	ND	Craniotomy	ND	ND
		75	M	Pituitary Macroadenoma	Skull base, Middle cranial fossa	ND	TSS	Bleeding CSF Leak	Subarachnoid bleeding, Intraventricular bleeding Hydrocephalus
Maolina et al	2019	23	F	Recurrent Nonfunctioning Pituitary Adenoma	Skull base, Middle cranial fossa	ICA	TSS	ND	ND
Mawk et al	1979	15	F	Sellar Mass, superior orbita fissura	Skull base, Anterior cranial fossa	MCA	Craniotomy	Bleeding	Diabetes Insipidus
		42	M	Supracellar Mass (PA Findings: Chromophobe Adenona)	Skull base, Middle cranial fossa	ND	Craniotomy	ND	Infarc Vascular
		23	M	Supracellar Mass, Third Ventricle And Upper Clivus	Skull base, Middle-posterior cranial fossa	ACA And Internal Carotis Artery	Craniotomy	ND	Diabetes insipidus
Nash et al	2016	48	F	Papillary Craniopharyngioma	Skull base, Anterior cranial fossa	Supraclinoid Carotid Arteries And Proximal A1/M1 Seg-Ments ACA)	TSS	Bleeding	Diabetes Insipidus
		49	F	Pituitary Mass (Adamantinomatous Craniopharyngioma)	Skull base, Middle cranial fossa	ND	TSS	Bleeding	Infarct
Nishioka et al	2001	41	F	Chromophobe Pituitary Adenoma	Skull base, Middle cranial fossa	ND	TSS	Bleeding	Intracapsular Hemorhage And Subarachnoid bleeding In Basal Cistern
Osterhage et al	2018	52	M	Giant Non-Functioning Pituitary Adenoma	Skull base, Middle cranial fossa	ND	TSS	Bleeding	Subarachnoid bleeding

		55	F	Rathke's Cleft Cyst	Skull base, Middle cranial fossa	ND	TSS	ND	Subarachnoid bleeding
		42	F	Rathke's Cleft Cyst / Granulation Tissue	Skull base, Middle cranial fossa	ND	TSS	ND	Subarachnoid bleeding
		56	F	Suprasellar Craniopharyngioma	Skull base, Middle cranial fossa	ND	TSS	ND	Subarachnoid bleeding-Intraventricular bleeding
Pan et al	2021	63	M	Chondrosarcoma Grade 2	Skull base, Anterior cranial fossa	ICA	Craniotomy	ND	Subdural bleeding-Subarachnoid bleeding
		53	F	Clinoidal Meningioma	Skull base, Anterior cranial fossa	Cavernous Segment Of ICA And Displaced The Left A1 And Middle Cerebral Artery	Craniotomy	Bleeding	ND
Popugaev et al	2011	45	M	Endosuprasellar Pituitary Adenoma	Skull base, Middle cranial fossa	ND	TSS	CSF Leak	Meningitis
		52	F	Giant Endosupracellar Pituitary Adenoma	Skull base, Middle cranial fossa	ND	TSS	CSF Leak	Meningitis
Puri et al	2012	59	M	Macroadenoma	Skull base, Middle cranial fossa	ICA	TSS	ND	Intratumoral Hemmorhage with Subarachnoid bleeding, Hydrocephalus
		36	F	Pituitary Macroadenoma	Skull base, Middle cranial fossa	ICA	TSS	Bleeding	Hemmorhage Suprasellar Aspect W/ Extension To Subarachnoid Basilar Cisterna
		66	M	Pituitary Macroadenoma	Skull base, Middle cranial fossa	Supraclinoid ICA	TSS	ND	Hemorrhage Within The Tumor Cavity Extending Into The Surrounding Subarachnoid Cisterns
Qi et al	2015	16	M	Schwannoma Left Part Of The Ventral Medulla Oblongata	Skull base, Posterior cranial fossa	ND	Craniotomy	Blood Leaking	No Complication
Rao et al	2013	10	F	Meningioma Cerebellopontine Angle Tumor	Skull base, Middle cranial fossa	ND	Craniotomy	ND	Hydrocephalus
Ricarte et al	2015	67	F	Craniopharyngioma	Skull base, Middle cranial fossa	ND	TSS	ND	ND

Salunke et al	2019	14	M	Craniopharyngiomas	Skull base, Middle cranial fossa	ND	TSS	ND	Intraventricular bleeding
Shao et al	2019	4	M	Large Arachnoid Cyst	Skull base, Anterior And Middle Cranial Fossae	ND	Craniotomy	ND	Increase Icp With Hygroma Post Op
Yassin et al	2020-2023	58	M	Ventricular Colloid Cyst	Skull base, Middle cranial fossa	ND	Endoscopic Resection Of The Colloid Cyst.	ND	Subarachnoid bleeding
Zada et al	2011	59	M	Macroadenoma	Skull base, Middle cranial fossa	ND	TSS	Intratumoral Hemmorhage, Subarachnoid bleeding	Hidrosephalus
		66	M	Macroadenoma	Skull base, Middle cranial fossa	B ACA	TSS	Intratumoral Hemmorhage, Subarachnoid bleeding	ND
		36	F	Pituitary Macroadenoma	Skull base, Middle cranial fossa	Anterior Com-Municating Artery Complex	TSS	Bleeding	Bleeding

ACA; Anterior Cerebral Artery, BILAT; Bilateral, CSF; Cerebrospinal Fluid, ECA; External Carotid Artery, ICA; Internal Carotid Artery, ICP; Intracranial Pressure, MCA; Middle Cerebral Artery, ND; Not Documented, PA; Pathology Findings, PCA; Posterior Cerebral Artery, Sx; Surgery, TSS; Transsphenoidal Surgery, VBA; Vertebrobasilar Artery.

Supplementary Table S2. Characteristics of vasospasm cerebral from study include.

First author	Time Between Craniotomy and Symptom Onset (days)	Vascular Vasospasme	Diagnostic Tool Vasospasm	Symptoms	
				Before Surgery	After Surgery (Vasospasm)
Afshari et al	8	B ICA, MCA, ACA	Angiography	2-month history of nausea and vomiting and daytime headache	left-sided hemiparesis with facial weakness and became drowsy
	5	L ICA, L ACA, R MCA	CT Angiography, Xenon CBF studies, TCD	decreased vision on the right side	Condition deteriorated
	6	R & L ICA	CT Angiography, Xenon CBF studies	history of visual change	Disoriented and developed right hemiparesis
Aoki et al	5	L ICA	CT Scan	severe headache and nuchal rigidity.	nausea and vomiting before lapsing into a coma
	6	R ICA,R MCA, R ACA	CTA	deterioration of vision, headache and loss of libido became blind in the right eye	Hemiprasesis
	30	ICA/MCA	Angiography		Hemiparesis
	15	VBA	Angiography	Progressive right paresthesias and left hemiparesis Dysphagia for 1 month Deficits in cranial nerves X and XI bilaterally	Obtunded
Bejjani et al	4	ACA/MCA	Angiography		Poorly Responsive
	11	ICA/MCA/ACA	Angiography		Lethargy
	1,67	L ACA	CT Scan		LLE Monoparesis
	1	R MCA	Angiography	ND	Hemiparesis
	2	B MCA/ACA	Angiography	ND	Lethargy
	1	L ICA/MCA	Angiography	ND	R UE monoparesis
	6	ACA/MCA	Angiography	ND	Asymptomatic
Bougaci et al	9	L ICA, L MCA and L ACA	Angiography	visual blurryness and loss	visual loss in the left eye, d a right hemiparesis with aphasia and paresthesia.

Budnick et al	13	L ICA, L MCA, L ACA	DSA, CTA, MRI/MRA, and TCD	Mild right homonymous hemianopsia	Acute dysarthria, anxiety, right-arm apraxia, gait instability, altered mental status and right hemiparesis
	7	B ACA, MCA, ICA	TCD, CTA	Vision loss, erectile dysfunction, diminished libido	Altered mental status and acute lethargy
Campt et al	6	R PCA, ICA	Angiogram Cerebral	amenorrhea, galactorrhea	left hemiparesis and n.6, n.7 palsy
	6	R MCA and R ACA	TCD	Subcontinuous headache, bitemporal field deficit	Disorientation and progressively worsening left hemiparesis
	5	R MCA	TCD	worsening, left hemiparesis	Progressively worsening left hemiparesis
Cervoni et al	7	B MCA	TCD	Subcontinuous headache, visual acuity deficit	Anisochoria and episodes of decerebration
	5	L MCA	TCD	Sudden onset of partial right motor epilepsy	Worsening left hemiparesis
	4	B MCA	TCD	Subcontinuous headache, severe visual impairment	A sudden onset of anisochoria and a motor response in decerebration.
Ecker et al	1	L MCA	Cerebral angiography	Sudden onset of a severe, leftsided headache 2 months before our evaluation. persistent nausea and intermittent problems finding words	The patient slowly developed word-finding difficulty and a mild right upperextremity drift
Eseonu et al	12	B ICA, L MCA	MRA, TCD	Headache, fatigue, blurred vision for several days. Physical examination revealed a bitemporal hemianopsia and a left optic nerve atrophy	Acute onset left sided paresthesia, left arm weakness, and expressive aphasia
Hashikata et al	0	B MCA	MRA	Worsening headache aggravated dementia, diplopia, right dysplasia, and gait disturbance for 6 months	Postoperative CT imaging on postoperative day 1 showed pneumocephalus and low-density areas the B insular and frontotemporal cortices, with no intracranial hemorrhage
Hyde-Rowan et al	2	B supraclinoid ICA	Angiography	Headaches, blurred vision on the left side, left-sided paracentral scotoma, visual acuity of 20/50 on the left side	Headache, lethargy, cardiorespiratory arrest, right-sided hemiparesis, aphasia
Kasliwal et al	13	L ICA	TCD Angiography	amenorrhea, galactorrhea and dull headache	R Sided hemiparesis, focal convulsions
LeuRox et al	7	R ICA, VBA	TCD,	12-month history of headache, left sensorineural hearing loss,	progressively confused and developed gait ataxia

				and gait ataxia.	
	7	L MCA and L ACA	TCD, CTA	progessive left frontal and retroorbital headache following a minor head injury Diplopia with upward gaze	Confusion, an expressive dysphasia, right-sided neglect, and motor weakness
	5	R ICA, MCA, ACA	Angiography	Headaches, diplopia, visual loss, amenorrhea	Left-sided hemiplegia, altered consciousness
Mansouri et al	7	L ACA/MCA	Angiography	Diplopia, bitemporal hemianopsia, Cushing's syndrome symptoms	Headache, vomiting, mild mixed aphasia
	1	L ICA, MCA	CT Perfusion	Decreased peripheral vision, visual blurring, headaches	Altered consciousness
Maolina et al	8	R ICA,R MCA, R ACA	CTA-MRI, DSA	ND	Mouth asymmetry accompanied by slurred speech and moderate weakness of the upper left limb.
	6	R MCA/R ACA	Angiography	headaches, recent weight gain, and primary aminorhea	left hemiparesis and a right third nerve palsy
Mawk et al	3	L ACA and L MCA	CT Scan and Carotid Angiography	disturbance of vision in the right eye and blind in the left eye	right hemiparesis
	10	R MCA/R ACA	ND	Progressive visual loss and decrease in libido	Left hemiplegia
Nash et al	11	ICA, B ACA/B MCA	Magnetic reso-nance angiography (MRA)	ND	episode of collapse, on day 11 with progressive dysphasia, lethargy, unsteadiness and hemiparesis
	1	ICA, B MCA	CT angiogram	deterioration in vision	5 days after surgery, with dysphasia and a reduced consciousness level
Nishioka et al	12	R MCA, R ACA, R ICA		Decreased libido, vision changes	Disorientation + L hemiparesis POD12 Aphasia + R-hemiparesis POD17
	0	L ACA and L MCA	CT/MRI TCD	Homonymous hemianopsia	4th postoperative day: somnolence, disturbed short-term-memory
Osterhage et al	2	R MCA, ACA dan ICA	CT/MRI	Headache, DM Insipidus	oculomotor palsy, afasia, brachiofacial hemiparesis
	2	ICA, PCA dan MCA B	CT/MRI	headache, dizziness, DM Insipidus	nausea, vomiting, headache, diplopia, 6 nerve palsy, afasia, distal paresis of left leg

Pan et al	0	R MCA dan VA	CT/MRI	Hidrocephal, Chiasm Syndrom, gait and speach disturbed	coma
	10	VA	angiography TCD	worsening abducens nerve palsy, cognitive decline, gait ataxia, and heavy snoring	complete recovery with only slight vision loss bilater ally
	6	L ACA dan MCA	CT angiogram	seizures, episodic diplopia, facial numbness, and left eye vision changes, without focal neurological deficits	partial nerve 3 palsy
Popugaev et al	4	B ICA	SCT-angiography TCD	ND	Febrile temperature and mild symptoms of meningism
	4	L MCA	TCD	ND	4th postoperative day delirium and respiratory insufficiency
Puri et al	5	B ICA, ACA, R MCA	MRI	ND	developed pro- gressively somnolent with left pupillary dilation
	9	B ACA	MRI	ND	agressively agitated and developed a dense expressive aphasia
	8	B ACA, L PCA	MRI	headaches and hypogonad- ism	deteriorated from altered mental status to obtundation, and dilated right pupil
Qi et al	3	ICA, BA	CT, MRI, MRA	Dizziness, projectile vomiting, unsteady walking, increased blood pressure	Unconscious state, hazy consciousness, coma, persistent vegetative state
Rao et al	7	B PCA, R MCA, L ACA	MRA, CTA, TCD	Headache, vomiting, gait instability, and decreased hearing in the right ear.	increased headache, nausea, vomiting, gait difficulty and bilateral leg weakness. right facial droop, right knee extensor 4+/5, left pronator drift, and left greater than right dysmetria
Ricarte et al	14	B ACA	TCD, CTA	Drowsiness Post-Surgery	Drowsiness, mild somnolence, apathy, residual paraparesis (grade 4/5)

Salunke et al	7	B ICA, ACA, MCA	CTA, DSA	retardation and progressive visual loss	Left hemiparesis + Visiol loss
Shao et al	12	B ICA, ACA, MCA	MRI, Catheter angiography, Transcranial Doppler (TCD)	Head tenderness and swelling, unsteady gait	Left hemiparesis with a facial droop,
Yassin et al	9	B MCA, BA	CTA	worst headache	Disartria + buckling out of legs
	5	B ACA	CT angiogram / DSA	visual loss and headache	de- teriorated vision in the left eye
Zada et al	7	B ACA, L PCA	CT angiogram	visual loss and hypogonadism	progressively somnolent with acute right pupillary dilation
	7	ACA	MR angiography	visual blurring, mild cognitive deficits, and symptoms of hypogonadism.	level of consciousness deteriorated, and his right pupil became acutely dilated

ACA; Anterior Cerebral Artery, B; Bilateral, BA; Basilar Artery, CTA; Computed Tomography Angiography, CT; Computed Tomography, DSA; Digital Subtraction Angiography, ICA; Internal Carotid Artery, L; Left, MCA; Middle Cerebral Artery, MRA; Magnetic Resonance Angiography, MRI; Magnetic Resonance Imaging, PCA; Posterior Cerebral Artery, R; Right, SCT; Spiral Computed Tomography, TCD; Transcranial Doppler, VA; Vertebral Artery.
